# Severe Legionnaires' Disease Complicated by Rhabdomyolysis and Clinically Resistant to Moxifloxacin in a Splenectomised Patient: Too Much of a Coincidence?

**DOI:** 10.1155/2015/793786

**Published:** 2015-11-22

**Authors:** Theocharis Koufakis, Ioannis Gabranis, Marianneta Chatzopoulou, Anastasios Margaritis, Maria Tsiakalou

**Affiliations:** ^1^Department of Internal Medicine, General Hospital of Larissa, 1 Tsakalof Street, 41221 Larissa, Greece; ^2^Department of Microbiology, General Hospital of Larissa, 41221 Larissa, Greece

## Abstract

We here report a case of Legionnaires' disease in a splenectomised patient, complicated by rhabdomyolysis and acute renal failure and characterized by a poor clinical response to moxifloxacin. Splenectomy is not included among the factors, typically associated with higher risk or mortality in patients with Legionellosis. However, our report is consistent with previous case reports describing severe* Legionella* infections in asplenic subjects. The possibility that functional or anatomic asplenia may be a factor predisposing to severe clinical course or poor response to therapy in patients with* Legionella* infection cannot be excluded, deserving further investigation in the future. More studies are required in order to clarify the underlying pathophysiological mechanisms that connect asplenia, immunological response to* Legionella*, and pathogen's resistance to antibiotics.

## 1. Introduction

*Legionellae* were firstly identified in 1976, as the causative agent of a pneumonia outbreak among subjects attending an American Legion convention in Philadelphia. They are intracellular Gram-negative parasites that replicate within alveolar macrophages and can cause two different clinical syndromes: pneumonia accompanied by multisystemic disease (known as Legionnaires' disease) and Pontiac fever, a viral-like, self-limited entity [1]. The organism has been isolated in natural aquatic habitats (freshwater streams and lakes, water reservoirs) and artificial sources (cooling towers, potable water distribution systems). Freshwater amoebae are believed to be the natural reservoir for* Legionella* [2]. Optimal growth temperature is 28–40°C, while organisms are inactive below 20°C and are murdered above 60°C.

In adults,* Legionella* causes 2–15% of community-acquired pneumonia cases that require hospitalization [3]. It is the second most common cause of serious pneumonia that needs admission in an Intensive Care Unit (ICU) [4]. Almost 80% of the reported cases are sporadic and the other 20% occur in outbreaks, mainly during summer and fall [5]. With regard to pediatric population,* Legionella* is considered as an uncommon cause of pneumonia, despite the fact that, according to serological studies, children are often exposed to the organism species [6].

Rhabdomyolysis is a clinical syndrome characterized by elevated serum concentrations of creatine phosphokinase (CPK) and myoglobinuria leading to renal dysfunction. It can be induced by several factors, such as trauma, ischemia, metabolic disorders, drugs, and viral and bacterial infections. Among bacteria that have been reported to cause rhabdomyolysis,* Legionella* species are believed to be the most common, followed by* Streptococcus* species,* Francisella tularensis*, and* Salmonella* species [7].

## 2. Case Presentation

**Figure 1:**
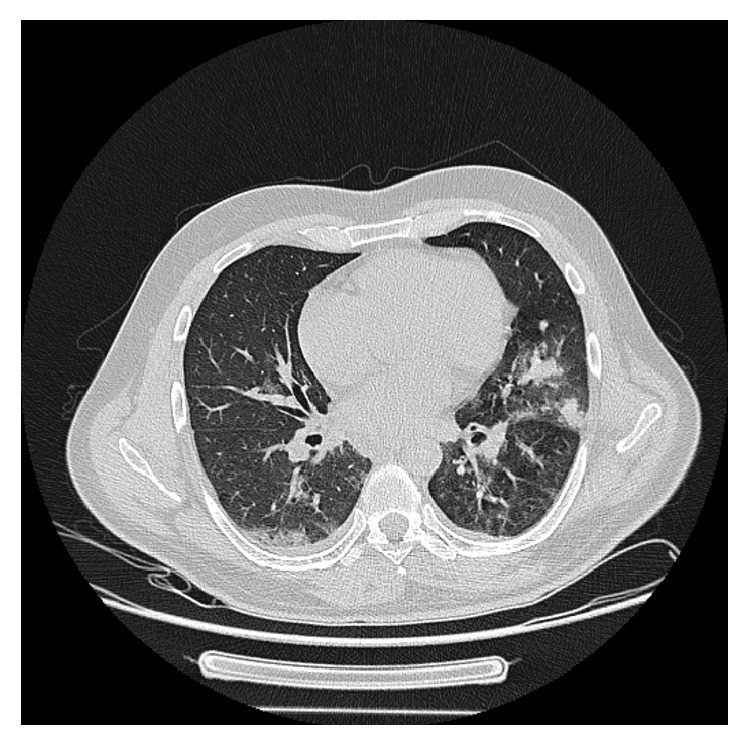
Computed tomography scan of the chest demonstrating diffuse bilateral opacities, mainly at the left side.

A 45-year-old male presented to the emergency department complaining about fever and fatigue for three days. He was a smoker (50 pack years) and a former heroin addict. Moreover, he had a history of splenectomy after a car crash at the age of five. The patient's main clinical and laboratory findings on admission were as follows: fever (39°C), sinus tachycardia (100 beats per minute), diffuse crackles heard on lung auscultation, hypoxemia (PO_2_ 59 mmHg), elevated inflammation markers (white cell count 18.1 × 10^3^ with 90% neutrophils, C reactive protein 16 mg/dL), abnormal renal function (urea 62 mg/dL; creatinine 1.85 mg/dL), and elevated creatine phosphokinase (CPK) serum levels (34371 units/L) (Table 1). He had a normal ECG, while his chest X-ray demonstrated diffuse bilateral opacities, mainly at the left side. Further evaluation with chest computed tomography confirmed the above findings (Figure 1). Urine, blood, and sputum samples were collected and sent for culture. Empiric therapy with moxifloxacin was started, as well as supportive care with intravenous fluids, alkalinization of urine, and oxygen.

Due to continuing anuria and further elevation of CPK and creatinine levels (maximum values during hospitalization 82026 units/L on day 3 and 10 mg/dL on day 7, resp.) (Table 1), the patient was subjected to hemodialysis 48 hours after admission. Hemodialysis sessions were continued daily for the next five days; however, the patient remained anuretic.

Urinary antigen for* Legionella* proved to be positive. Still, no pathogenic organism was isolated from the blood, urine, and respiratory cultures. Further laboratory evaluation revealed that the patient was positive for hepatitis C virus (HCV) antibody, while other serological tests for common bacteria and other viruses were negative. Patient's exposure to any source of the microorganism could not be documented.

Seven days after admission, he presented tachypnea (40 breaths per minute) and further deterioration of hypoxemia (PO_2_ 50 mmHg). He was subsequently shifted to the ICU, where he was supported by noninvasive ventilation (NIV) and continuous hemofiltration. Because of fever's persistence and continuing deterioration of patient's clinical status, azithromycin was added to the antibiotic scheme. Initiation of azithromycin was followed by a rapid improvement of both clinical condition and laboratory tests. After 2 days in the ICU, the patient was transferred to the floor, where sessions of conventional hemodialysis were continued. After having restored satisfactory diuresis and normal CPK and creatinine blood levels and since no recurrence of fever was observed, he was discharged 30 days after admission. In his follow-up visits, he remained in good health and his laboratory tests were all within the normal values.

## 3. Discussion

Mortality rate in patients with Legionnaires' disease varies between 5 and 80% [8]. The following factors have been associated with high mortality: age (infants and elderly), predisposing underlying conditions (such as chronic lung disease, immunodeficiency, malignancies, end-stage renal disease, and diabetes mellitus), nosocomial acquisition, and delayed initiation of specific antimicrobial therapy [9].

Diagnosis is based mainly on the isolation of the pathogen from infected tissues and fluids, while imaging, histopathological, and other laboratory methods are of limited use, due to the fact that* Legionella* infection does not present with specific signs and symptoms. However,* Legionella *spp. do not grow on standard microbiology media and are usually not detected by blood culture or Gram stain or culture of sputum [10]. Urine antigen test is a rapid, practical, and inexpensive method for the diagnosis of the disease, characterized by specificity that almost reaches 100% [11]. The above explain the fact that the availability of tests for* Legionella* antigen in the urine resulted in a limited use of cultures and serological studies [12]. The primary disadvantage of urinary testing is that it detects only* Legionella pneumophila* serogroup 1.

Fluoroquinolones (such as moxifloxacin) and newer macrolides (such as azithromycin) are generally considered as the antibiotic agents of choice, regarding the treatment of* Legionella* pneumonia. Combined treatment is believed to be superior to monotherapy in cases of severe clinical disease or in immunosuppressed subjects [13]. In general,* Legionella* species are susceptible to antibiotics of choice; still, resistant strains have been described, especially in cases treated with rifampin [13]. The duration of treatment, with moxifloxacin or azithromycin, should be 7–10 days in uncomplicated cases and should reach 21 days in severe cases or in immunocompromised hosts [14].

The first report that associated* Legionella* and rhabdomyolysis was published in 1980 by Posner et al. [15] and since then, the above relationship has been well established. The exact mechanism of muscle injury caused by* Legionella* is still unclear. However, release of an endotoxin or exotoxin that causes rhabdomyolysis and direct bacterial invasion seem to be the most probable mechanisms [7]. High morbidity (57% of cases with acute renal failure) and mortality (death in 38% of cases) are linked with bacterial causes of rhabdomyolysis [7].

*Legionella* is an intracellular pathogen; therefore, cell-mediated immunity is believed to be the major host defence mechanism against the infection. Several studies have demonstrated a higher incidence of* Legionella* infection in patients under glucocorticoids and immunosuppressive drugs, transplant recipients, and those suffering from hairy cell leukemia [10]. Interestingly, HIV infection does not seem to predispose to the development of Legionnaires' disease [16]. Similarly, splenectomy is not included among the factors, typically associated neither with higher risk for developing Legionellosis, nor with higher mortality, in hospitalised patients with community-acquired Legionnaires' disease [17]. Asplenic individuals mainly have impaired humoral immunity and B-lymphocyte function and, to a smaller degree, some decrease in cell-mediated immunity [18]. Our patient had been subjected to splenectomy. We consider that this fact contributed to the severe clinical course, characterized by rhabdomyolysis and acute renal failure, demanding the combination of two antibiotics for the control of the infection. Our report is consistent with previous case reports [18, 19] describing severe* Legionella* infections in splenectomised subjects.

The observed* Legionella* resistance to moxifloxacin in our case is generally uncommon. Still, it was a clinical finding that could not be confirmed by laboratory methods. After inhalation* Legionella pneumophila* is taken up by alveolar macrophages within which it is able to survive and replicate. The particular property is believed to act as a major immune defence and antimicrobial treatment evasion strategy. Observational studies of clinical response to therapy have been valuable due to the lack of standardised susceptibility testing and the pathogen's unpredictable in vivo response to treatment, because of its intracellular life cycle. Between the two most commonly used antibiotic classes, that is, macrolides and quinolones, that achieve high intracellular concentrations, observational studies suggest that quinolones are more likely to achieve a favourable outcome in terms of patient survival and length of hospitalization [20–22]. There is, however, in the same studies, a consistently small proportion of patients that fails to respond to fluoroquinolone treatment. Molecular evidence suggests that therapeutic failure could be the result of mutant selection during therapy that code for low-affinity DNA topoisomerases [23]. This process is clinically accompanied by a rise of the antibiotic MIC against the pathogen and a higher likelihood of therapeutic failure. Differently, older studies pointed toward late onset of treatment as the main cause of therapeutic failure in Legionnaires' disease [24]. This was not the case in our patient, given that the combination of rhabdomyolysis and pneumonia made us suspect in time a probable* Legionella* infection and moxifloxacin was started upon presentation. It is our belief that the rapid initiation of treatment, in addition to the early substitution of renal function, resulted in the good outcome.

In conclusion,* Legionella* pneumonia should always be suspected in patients presenting with rhabdomyolysis, fever, and an abnormal chest X-ray. Early start of appropriate treatment is important and can be proved lifesaving. The possibility that functional or anatomic asplenia may be a factor predisposing to severe clinical course or poor response to therapy in patients with* Legionella* infection cannot be excluded, deserving further investigation in the future. More studies are required in order to clarify the underlying pathophysiological mechanisms that connect asplenia, immunological response to* Legionella*, and pathogen's resistance to antibiotics.

## Figures and Tables

**Table 1:** Patient's main laboratory findings on admission and discharge, as well as maximum values observed during hospitalization. Interestingly, the decrease in number of WBC was observed after initiation of azithromycin on day 7, suggesting a probable *Legionella* resistance to moxifloxacin.

Parameter in blood (units)	Admission	Maximum value during hospitalization (day)	Discharge
White blood cell (10^3^/*μ*L)	18.1	24.5 (7)	13.2
Creatine phosphokinase (IU/L)	34371	82026 (3)	150
Creatinine (mg/dL)	1.85	10.0 (7)	0.95
Urea (mg/dL)	62	195 (7)	26
